# Causal relationship between breakfast skipping and bone mineral density: a two-sample Mendelian randomized study

**DOI:** 10.3389/fendo.2023.1200892

**Published:** 2023-11-07

**Authors:** Jinsheng Yu, Chen Zhuang, Wenxuan Guo, Xing Zhou, Yixuan Chen, Likang Wang, Wenkai Li, Yiwen Zhu, Rujie Zhuang, Kun Tian

**Affiliations:** ^1^ The First School of Clinical Medicine, Zhejiang Chinese Medical University, Hangzhou, Zhejiang, China; ^2^ Alberta Institute, Wenzhou Medical University, Wenzhou, China; ^3^ Orthopedic Department, The First Affiliated Hospital of Zhejiang Chinese Medical University (Zhejiang Provincial Hospital of Traditional Chinese Medicine), Hangzhou, China

**Keywords:** genetics, Mendelian randomization, causal relationship, bone mineral density, breakfast skipping

## Abstract

**Objective:**

To explore the causal association between breakfast skipping and bone mineral density (BMD) through two-sample Mendelian randomisation (MR) analysis.

**Methods:**

A two-sample MR approach was adopted to explore the causal relationship of breakfast skipping with BMDs (across three skeletal sites and five age groups). Publicly available genome-wide association study summary data were used for MR analysis. We used five methods to estimate the causal associations between breakfast skipping and BMDs: inverse-variance weighting (IVW), MR-Egger, weighted median, simple mode, and weighted mode. IVW was used for the main analysis and the remaining four methods were used as supplementary analyses. The heterogeneity of the MR results was determined using IVW and MR-Egger methods. The pleiotropy of the MR results was determined using MR-Egger intercept. Furthermore, a leave-one-out test was performed to determine whether the MR results were affected by a single nucleotide polymorphism.

**Results:**

With the IVW method, we did not find any causal relationship between breakfast skipping and forearm, femoral neck, and lumbar spine BMD. Subsequently, when we included BMD data stratified by five different age groups in the analysis, the results showed that there was no apparent causal effect between breakfast skipping and age-stratified BMD. This finding was supported by all four supplementary methods (P > 0.05 for all methods). No heterogeneity or horizontal pleiotropy was detected in any of the analyses (P > 0.05). The leave-one-out tests conducted in the analyses did not identify any single nucleotide polymorphism that could have influenced the MR results, indicating the reliability of our findings.

**Conclusion:**

No causal effect was found between breakfast skipping and BMD (across three skeletal sites and five age groups).

## Introduction

Ageing-related disorders have become more prevalent in contemporary society because of advancements in healthcare, changes in socioeconomic conditions, and improvements in lifestyle. As a result, there has been a significant improvement in life expectancy (1). Osteoporosis is a common condition that affects the musculoskeletal system, causing a reduction in bone mass and degradation of bone microarchitecture. This, in turn, increases the risk of individuals developing pathological fractures because of decreased bone density (2). Currently, the method for diagnosing osteoporosis is by evaluating a patient’s bone mineral density (BMD) through dual energy X-ray absorptiometry (DXA) assessment, which is also the gold standard for defining osteoporosis (3). Osteoporosis is more common in women than men, with a prevalence of 29.9% in women aged 50 years or older, which is 13.9% higher than that in men (4). An estimated $25 billion is expected to be spent on osteoporosis-related pathological fractures in the United States in 2025 (5). The primary approach for treating osteoporosis at present is through medication, including bisphosphonates, calcitonin, oestrogen, and oestrogen receptor agonists. However, these medications have limited efficacy in treating the condition and can cause significant adverse reactions (6). Therefore, it is important to recognise the risk factors that contribute to osteoporosis, screen individuals who are at risk, and facilitate prevention and early intervention as necessary. Many studies have demonstrated factors that are associated with the disease, such as older age, sex, as well as a diverse range of clinical, medical, behavioural, nutritional, and genetic factors (3, 5, 7, 8).

Food intake timing is a contributing factor to the risk of chronic diseases (9, 10). Breakfast, as the first meal of the day, plays an important role in providing essential nutrients and regulating metabolism. According to research, individuals who do not eat breakfast during both childhood and adulthood are more likely to develop various chronic diseases, including hypertension, cardiovascular metabolic disorders, insulin resistance, and diabetes, in adulthood. By contrast, individuals who have breakfast during both periods have a lower risk (11). In a report targeting adult males in Japan, research findings have indicated that adult males who regularly eat breakfast have the highest bone density, followed by those who occasionally eat breakfast, while the group who rarely eat breakfast have the lowest bone density (12). Another 3-year prospective study supports this viewpoint, indicating that skipping breakfast is not only significantly associated with a decline in bone density but also with changes in bone structure. This correlation exists in both male and female individuals (13). However, we need to approach the results of observational studies with caution, as they are susceptible to confounding factors and reverse causation. Randomised controlled trials have a higher level of evidence compared with other study designs, but they require more resources in terms of manpower, materials, and finances, and can be difficult to implement.

Mendelian randomisation (MR) is an analytical method that investigates causal relationships between exposure and outcome by using genetic variables as instrumental variables (IVs). This analytical approach follows Mendel’s law of inheritance, in which alleles are randomly distributed during meiosis, substantially reducing interference from confounding factors. Furthermore, since diseases occur after genetic variations, and the temporal sequence cannot be reversed, MR analysis is not influenced by reverse causality when exploring causal relationships between exposure and outcome. Its mechanism is similar to that of randomised controlled trials (14, 15), making this genetic-variable-based analytical method capable of yielding more reliable conclusions (16). Previous MR studies have demonstrated causal effects of factors such as fasting glucose (17), low-density lipoprotein cholesterol (18), and central obesity (19) on the development of BMD. However, an MR study on the causal effect of breakfast skipping on BMD has not been conducted.

In this study, a two-sample MR approach was used to estimate the causal effects of breakfast skipping on site-specific and age-specific BMDs.

## Methods

### Study design

**Figure 1:**
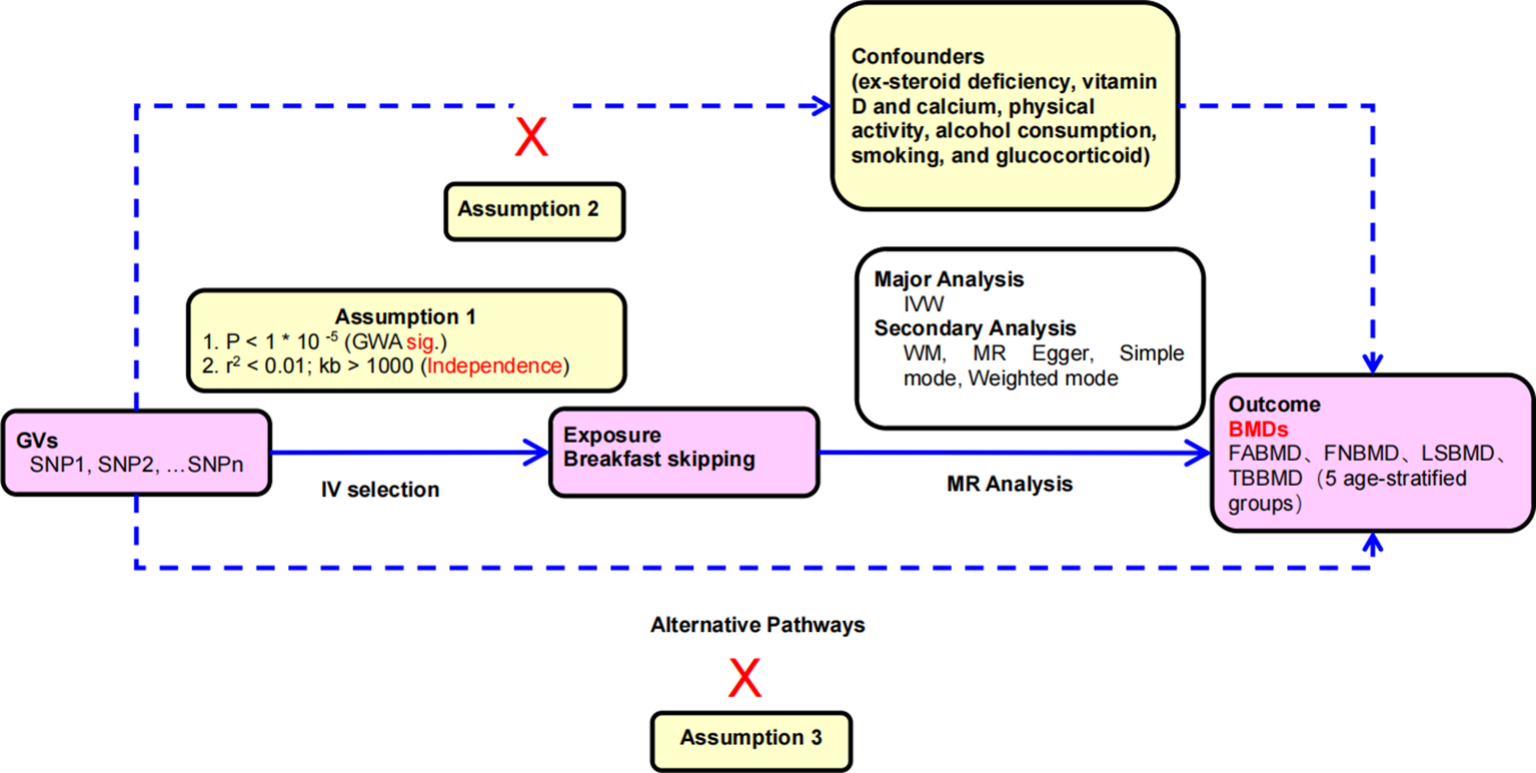
Principles of mendelian randomization study: (1) The relevance assumption: there is a strong correlation between the instrumental variable and the exposure factor being studied. (2) The independence assumption: the instrumental variable should not be associated with any potential confounder; (3) The exclusion restriction assumption: the instrumental variable should influence the outcome risk merely through the exposures, not via any alternative pathway.

Specifically, in MR analysis, as shown in **Figure 1**, effective IVs must adhere to three key assumptions. Firstly, only genetic variants (single nucleotide polymorphisms, SNPs) strongly associated with the exposure (breakfast skipping) can be used as IVs. Secondly, genetic variants should not be correlated with any potential confounding factors to ensure the reliability of the MR analysis. Thirdly, genetic variants must influence the outcome (BMDs) solely through their effect on the exposure and should not impact the outcome through any other pathways. Building upon these assumptions, we estimated the causal relationship between breakfast skipping and forearm BMD (FABMD), femoral neck BMD (FNBMD), lumbar spine BMD (LSBMD), and across five age groups (0–15 years, 15–30 years, 30–45 years, 45–60 years, and over 60 years).

### Data sources

We chose the most comprehensive genome-wide association study (GWAS) data on breakfast skipping (20). Dietary data were collected from 193,860 participants, including 106,284 female participants (accounting for approximately 54% of the total sample) using Oxford WebQ (21), a web-based 24-hour diet recall. The participants had an average age of 56.8 years and an average body mass index of 26.9 kg/m2. This method required participants to report their dietary intake over the past 24 hours. Breakfast skipping was assessed with the use of data from up to five web-based 24-hour diet recalls. Participants were asked, “Did you eat breakfast yesterday? This could be at any time. Please choose Yes or No”. Responses from all completed recalls (up to five times) were considered. The responses were categorised into three groups: breakfast skipping (always responded “No”), sometimes breakfast skipping (sometimes responded “Yes”), and breakfast consumers (always responded “Yes”). Full summary statistics for the breakfast skipping (N = 193,860) GWAS are available for download from the Musculoskeletal KP genetic association datasets (https://msk.hugeamp.org/datasets.html).

The forearm, femoral neck, and lumbar spine are the three skeletal sites most commonly used for DXA-based BMD measurement when diagnosing and screening for osteoporosis. The GWAS data for BMDs was downloaded from the Genetic Factors for Osteoporosis Consortium website (http://www.gefos.org/). The data provide bone density measurements for the forearm, femoral neck, and lumbar spine, with sample sizes of 8,143 (FABMD), 32,735 (FNBMD), and 28,498 (LSBMD). These data are from the largest GWAS study to date on DXA-measured BMD and represent the most comprehensive dataset available on this topic (22).

In addition, we obtained total body BMD (TBBMD) data for five age groups (0–15 years, 15–30 years, 30–45 years, 45–60 years, and over 60 years) from a large GWAS meta-analysis (23), encompassing data for 66,628 individuals (0–15 years: n = 11,807; 15–30 years: n = 4,180; 30–45 years: n = 10,062; 45–60 years: n = 18,805; and over 60 years: n = 22,504). In this study, all individuals (except for the age-stratified BMD data) belong to the European population, and the age-stratified data were provided by individuals of European ancestry (86%) and mixed backgrounds (14%).

### Instrument selection

First, we extracted SNPs strongly correlated with the exposure (breakfast skipping), using a screening criterion of (P < 5 × 10^−8^) (24). Second, to avoid a linkage disequilibrium among the included SNPs, we used the “clump” function to select independent SNPs (r^2^ <0.001 and windows size >10,000 kb) as IVs, which ensured reliability of the analysis results (25). After screening, only six SNPs were identified, so we relaxed the criteria to 1 × 10^−5^, R^2^ < 0.01, and window size = 1,000 kb for selecting breakfast skipping-related IVs. Third, we manually reviewed SNPs that met the above criteria using the PhenoScanner database, excluding SNPs directly related to osteoporosis and SNPs associated with confounding factors (risk and protective factors) for osteoporosis. Confounding factors include but are not limited to sex-steroid deficiency (26, 27), vitamin D and calcium (28, 29), physical activity (30), alcohol consumption (31, 32), smoking (33), and glucocorticoid (34)). Fourth, after the criteria-based screening, qualified SNPs were used to extract outcome information. During this process, if SNP information was missing, we did not use proxy functions. Additionally, we further screened the extracted SNPs to remove those with non-concordant alleles and palindromic SNPs with ambiguous strands (35, 36). Fifth, to enhance the reliability of the MR analysis results, we calculated the F-statistic of each SNP using the formula (37), and if the F-statistic was greater than 10, the results were less likely to be influenced by weak IVs (37). Finally, we retained the SNPs that met the criteria as IVs for the subsequent MR analysis.

### Statistical analysis

We employed five methods for performing MR analysis to investigate the causal relationship between breakfast skipping and BMD. The inverse-variance weighting (IVW) (38) method assumes that all SNPs are valid instrumental variables and that there is no correlation between each SNP. In this scenario, the IVW method can provide the most accurate causal estimates (39), while the methods MR-Egger (40), weighted median (41), simple mode (42), and weighted mode (43) are considered supplementary analysis methods. In addition, we conducted heterogeneity, pleiotropy, and sensitivity analyses to assess whether heterogeneity and pleiotropy would impact our MR results. First, to quantitatively evaluate the degree of heterogeneity among individual SNPs, we calculated the Q-statistics and I^2^ values (44). The “leave-one-out” approach was used to identify potentially influential SNPs. This involved performing the MR analysis multiple times, each time leaving out a different SNP (40). Subsequently, we employed the “mr_pleiotropy_test” function from the R TwoSampleMR package to examine the pleiotropy of our effect estimates using the MR-Egger intercept method. Lastly, to obtain outlier-adjusted estimates of causal associations, we employed the MR-PRESSO (Pleiotropy RESidual Sum and Outlier) analysis. This involved removing one or more pleiotropic outlying SNPs and reconducting MR analyses (45). All statistical analyses were performed using R (version 4.2.2) using the Two-Sample MR package (22), with P values < 0.05 regarded as statistically significant.

## Results

### Eligible SNPs

SNPs that did not meet the criteria, including those with linkage disequilibrium or MAF < 0.01, palindromic SNPs, and confounder-related SNPs, were excluded. The remaining SNPs that met the criteria can be found in Additional file 1. Afterward, we extracted the SNPs and their corresponding statistical data that were associated with the outcome. We selected 15, 14, 14, 19, 18, 20, 20, and 20 SNPs as instrumental variables for the causal analyses between breakfast skipping and FABMD, FNBMD, LSBMD, TBBMD (0–15 years), TBBMD (15–30 years), TBBMD (30–45 years), TBBMD (45–60 years), and TBBMD (over 60 years). Detailed information is provided in Additional file 2.

### Causal effects of breakfast skipping on BMDs by site

**Figure 2:**
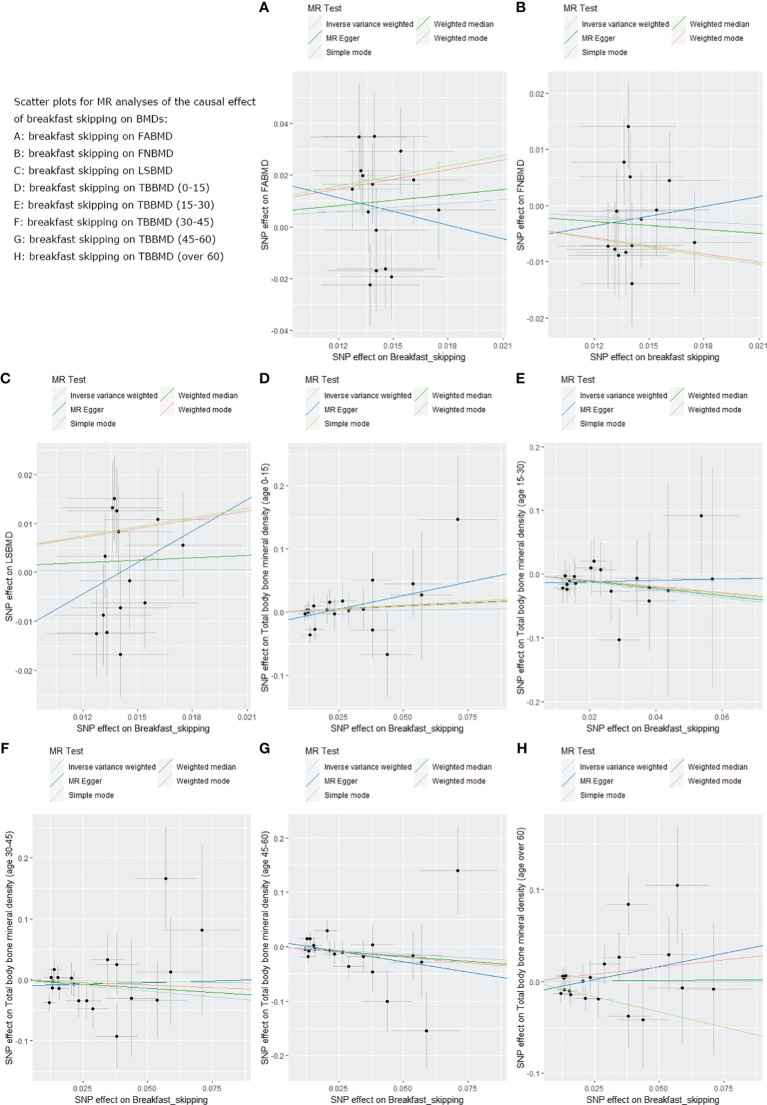
Scatter plots for the MR analysis, which explore the causal effect of breakfast skipping on BMDs. **(A)**: The scatter plot for breakfast skipping on FABMD. **(B)**: The scatter plot for breakfast skipping on FNBMD. **(C)**: The scatter plot for breakfast skipping on LSBMD. **(D)**: The scatter plot for breakfast skipping on TBBMD (0-15). **(E)**: The scatter plot for breakfast skipping on TBBMD (15-30). **(F)**: The scatter plot for breakfast skipping on TBBMD (30-45). **(G)**: The scatter plot for breakfast skipping on TBBMD (45-60). **(H)**: The scatter plot for breakfast skipping on TBBMD (over 60).

**Figure 3:**
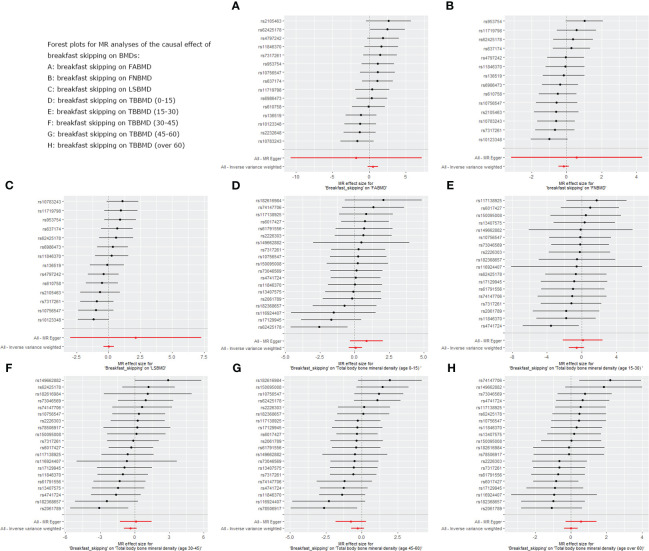
Forest plot for MR analysis of single and summarized SNPs effects on relationship between “breakfast skipping” and “BMDs”. **(A)**: The forest plot for breakfast skipping on FABMD. **(B)**: The forest plot for breakfast skipping on FNBMD. **(C)**: The forest plot for breakfast skipping on LSBMD. **(D)**: The forest plot for breakfast skipping on TBBMD (0-15). **(E)**: The forest plot for breakfast skipping on TBBMD (15-30). **(F)**: The forest plot for breakfast skipping on TBBMD (30-45). **(G)**: The forest plot for breakfast skipping on TBBMD (45-60). **(H)**: The forest plot for breakfast skipping on TBBMD (over 60).

**Figure 4:**
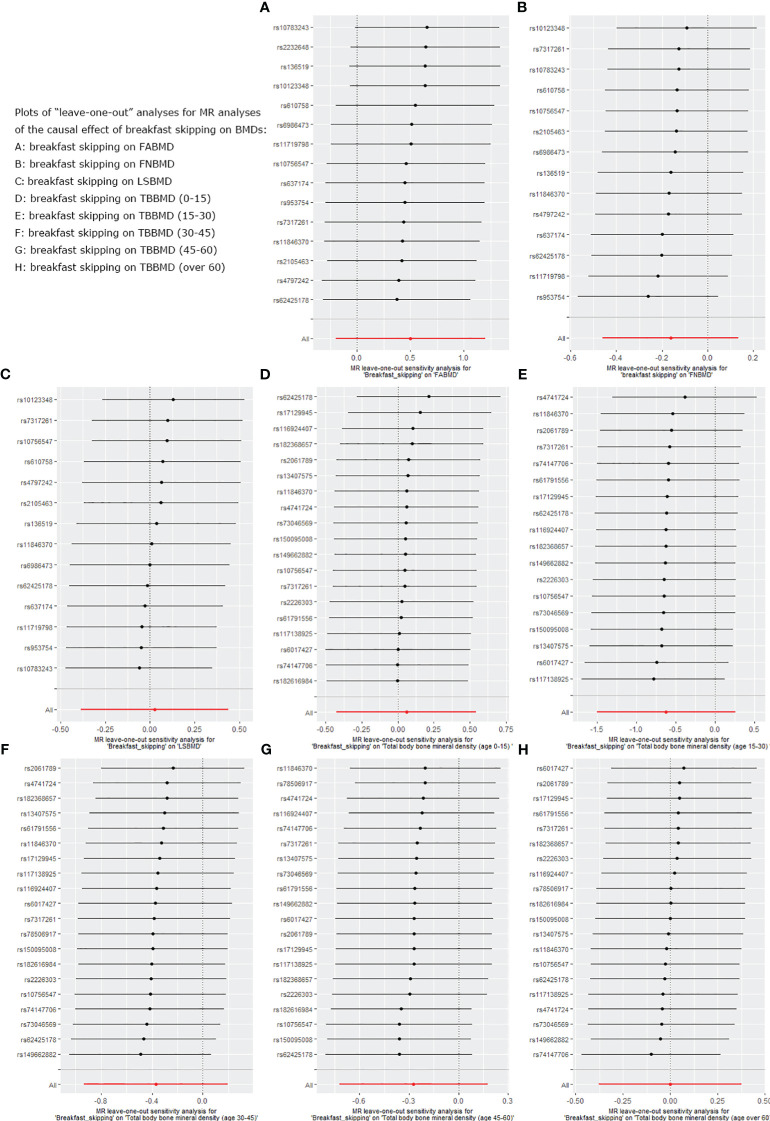
Leave-one-out plot for sensitivity analysis of single SNP effect on “breakfast skipping” to “BMDs” MR results. **(A)**: The “leave-one-out” sensitivity test for breakfast skipping on FABMD. **(B)**: The “leave-one-out” sensitivity test for breakfast skipping on FNBMD. **(C)**: The “leave-one-out” sensitivity test for breakfast skipping on LSBMD. **(D)**: The “leave-one-out” sensitivity test for breakfast skipping on TBBMD (0-15). **(E)**: The “leave-one-out” sensitivity test for breakfast skipping on TBBMD (15-30). **(F)**: The “leave-one-out” sensitivity test for breakfast skipping on TBBMD (30-45). **(G)**: The “leave-one-out” sensitivity test for breakfast skipping on TBBMD (45-60). **(H)**: The “leave-one-out” sensitivity test for breakfast skipping on TBBMD (over 60).

We used five different analytical methods to explore the causal relationship between breakfast skipping and BMDs. The results of these methods are presented in **Tables 1**, **2**. In the analysis, the IVW method did not find any proof to suggest that breakfast skipping causes changes in BMDs (FABMD: β = 0.502, Se = 0.355, P = 0.158; FNBMD: β = −0.162, Se = 0.151, P = 0.284; LSBMD: β = 0.026, Se = 0.211, P = 0.901). **Figures 2A–C** present scatter plots of the MR analysis for the causal relationship between breakfast skipping and FABMD, FNBMD, and LSBMD, respectively. In **Figures 3A–C**, the causal relationships between breakfast skipping and FABMD, FNBMD, and LSBMD, respectively, are presented in forest plots. In the figures, we could observe the causal effects of individual SNPs as well as the overall SNPs effects. Finally, we present the results of the leave-one-out analysis in **Figures 4A–C**. During this process, we did not identify any single SNP with a strong effect that would influence the overall outcome, demonstrating the robustness and reliability of our findings.

**Table 1:** MR estimates from different methods of assessing the causal effect of breakfast skipping on BMDs by site.

Exposure	Outcome	N_snp	Method	beta	SE	p_value
Breakfast skipping	**FABMD**	15	MR Egger	-1.761	4.566	0.705
		15	Weighted median	0.686	0.433	0.113
		15	Inverse variance weighted	0.502	0.355	0.158
		15	Simple mode	1.316	0.892	0.162
		15	Weighted mode	1.239	0.895	0.187
	**FNBMD**	14	MR Egger	0.575	1.909	0.768
		14	Weighted median	-0.234	0.210	0.263
		14	Inverse variance weighted	-0.162	0.151	0.284
		14	Simple mode	-0.497	0.361	0.192
		14	Weighted mode	-0.476	0.358	0.207
	**LSBMD**	14	MR Egger	2.140	2.622	0.430
		14	Weighted median	0.165	0.245	0.498
		14	Inverse variance weighted	0.026	0.211	0.901
		14	Simple mode	0.626	0.574	0.295
		14	Weighted mode	0.598	0.540	0.288

**Table 2:** MR estimates from different methods of assessing the causal effect of breakfast skipping on BMDs at different ages.

Exposure	Outcome	N_snp	Method	beta	SE	p_value
Breakfast skipping	**TBBMD (0-15)**	19	MR Egger	0.855	0.602	0.174
		19	Weighted median	0.195	0.331	0.554
		19	Inverse variance weighted	0.060	0.246	0.806
		19	Simple mode	0.229	0.542	0.676
		19	Weighted mode	0.192	0.537	0.725
	**TBBMD (15-30)**	18	MR Egger	0.111	1.155	0.924
		18	Weighted median	-0.560	0.591	0.342
		18	Inverse variance weighted	-0.622	0.450	0.166
		18	Simple mode	-0.508	1.051	0.634
		18	Weighted mode	-0.478	1.037	0.650
	**TBBMD (30-45)**	20	MR Egger	0.106	0.697	0.880
		20	Weighted median	-0.273	0.391	0.483
		20	Inverse variance weighted	-0.369	0.288	0.200
		20	Simple mode	-0.052	0.695	0.941
		20	Weighted mode	-0.165	0.629	0.795
	**TBBMD (45-60)**	20	MR Egger	-0.759	0.536	0.174
		20	Weighted median	-0.353	0.281	0.209
		20	Inverse variance weighted	-0.272	0.228	0.232
		20	Simple mode	-0.393	0.460	0.403
		20	Weighted mode	-0.393	0.455	0.398
	**TBBMD (over 60)**	20	MR Egger	0.570	0.439	0.211
		20	Weighted median	0.023	0.260	0.929
		20	Inverse variance weighted	-0.001	0.191	0.994
		20	Simple mode	-0.662	0.534	0.230
		20	Weighted mode	0.311	0.472	0.517

### Causal effects of breakfast skipping on BMDs at different ages

In the causal analysis of breakfast skipping and age-stratified BMDs, no significant causal relationship was found between breakfast skipping and BMD in five different age groups. The IVW method, as the primary analytical approach, yielded the following results (TBBMD 0–15 years: β = 0.060, Se = 0.246, P = 0.806; TBBMD 15–30 years: β = −0.622, Se = 0.450, P = 0.166; TBBMD 30–45 years: β = −0.369, Se = 0.288, P = 0.200; TBBMD 45–60 years: β = −0.272, Se = 0.228, P = 0.232; TBBMD over 60 years: β = −0.001, Se = 0.191, P = 0.994). The results from the remaining four supplementary analysis methods supported the findings of the IVW method. In **Figures 2D–H**, scatter plots are provided for the causal analysis of breakfast skipping and age-stratified BMDs. **Figures 3D–H** present forest plots for individual SNP effect analysis. Similarly, in the leave-one-out analysis, as shown in **Figures 4D–H**, no SNP with a strong driving force effect on the overall outcome was found.

As shown in **Table 3**, no significant pleiotropy and heterogeneity were found in the analysis: breakfast skipping−FABMD: IVW, p = 0.130; MR-Egger, p = 0.105; MR-Egger intercept = 0.032, p = 0.627; breakfast skipping−FNBMD: IVW, p = 0.434; MR-Egger, p = 0.368; MR-Egger intercept = −0.010, p = 0.705; breakfast skipping−LSBMD: IVW, p = 0.129; MR-Egger, p = 0.120; MR-Egger intercept = −0.030, p = 0.434; breakfast skipping−TBBMD 0–15 years: IVW, p = 0.617; MR-Egger, p = 0.698; MR-Egger intercept = −0.016, p = 0.166; breakfast skipping−TBBMD 15–30 years: IVW, p = 0.975; MR-Egger, p = 0.972; MR-Egger intercept = −0.014, p = 0.500; breakfast skipping−TBBMD 30–45 years: IVW, p = 0.381; MR-Egger, p = 0.355; MR-Egger intercept = −0.009, p = 0.461; breakfast skipping−TBBMD 45–60 years: IVW, p = 0.188; MR-Egger, p = 0.193; MR-Egger intercept = 0.010, p = 0.329; breakfast skipping−TBBMD over 60 years: IVW, p = 0.348; MR-Egger, p = 0.413; and MR-Egger intercept = 0.012, p = 0.168.

**Table 3:** Pleiotropy and heterogeneity test of the breakfast skipping from BMDs GWAS.

exposure	outcome	Pleiotropy test			Heterogeneity test					
		MR-Egger			MR-Egger			IVW		
		Intercept	SE	P	Q	Q_df	Q_pval	Q	Q_df	Q_pval
Breakfast skipping	FABMD	0.032	0.064	0.627	19.61	13	0.105	19.98	14	0.130
	FNBMD	-0.010	0.027	0.705	13.00	12	0.368	13.16	13	0.434
	LSBMD	-0.030	0.037	0.434	17.83	12	0.120	18.80	13	0.129
	TBBMD (0-15)	-0.016	0.011	0.166	13.55	17	0.698	15.63	18	0.617
	TBBMD (15-30)	-0.014	0.021	0.500	7.03	16	0.972	7.50	17	0.975
	TBBMD (30-45)	-0.009	0.013	0.461	19.60	18	0.355	20.21	19	0.381
	TBBMD (45-60)	0.010	0.010	0.329	22.93	18	0.193	24.21	19	0.188
	TBBMD (over 60)	0.012	0.008	0.168	18.65	18	0.413	20.79	19	0.348

## Discussion

To the best of our knowledge, this is the first MR study on the causal relationship between breakfast skipping and BMDs. We utilised data from large GWAS and the UK Biobank to assess the potential causal effect between the two. Our study did not observe a causal effect of breakfast skipping on BMDs in the forearm, femoral neck, and lumbar spine. Subsequently, we conducted further analyses stratified by age groups, and the results indicated that there was no significant causal effect of breakfast skipping on BMDs across the five age groups.

Breakfast is an indicator of a health-promoting lifestyle and behaviour, and breakfast skipping is considered a subclinical eating disorder (46). It is frequently evaluated in certain epidemiological studies (47, 48). The heritability of this behaviour as a genetic trait ranges from 41% to 66% (49). Moreover, it is a risk factor for a range of diseases, such as obesity, type 2 diabetes, cardiovascular disease, and metabolic syndrome (50–52). Behavioural studies related to this indicate that smokers are more prevalent among breakfast skippers, and the likelihood of smoking increases with the frequency of breakfast skipping during the week (53). Additionally, breakfast skippers tend to consume higher alcohol levels, with an average daily alcohol intake of 20.5 grams for breakfast skippers compared with 8.6 grams for breakfast eaters (54, 55).

In a study related to the correlation between breakfast skipping and BMDs, Kuroda et al. (56) conducted an observational study comparing the relationship between breakfast frequency and BMDs. The results showed that individuals who skipped breakfast 1–2 times a week had lower hip BMD than those who ate breakfast every day, but the results were not statistically significant. However, individuals who skipped breakfast at least three times a week had significantly lower hip BMD than those who skipped breakfast 1–2 times a week and those who ate breakfast every day (p=0.071, p=0.007, respectively). However, these differences were not significant in the comparison of lumbar spine BMD. It is noteworthy that all the participants involved in this study were female. In another study regarding the relationship between skipping breakfast and bone density, Nagata et al. (13) found a significant association between breakfast skipping and reduced lumbar spine BMD in men. At the same time, this study supports the viewpoint of Kuroda et al., indicating that in the female population, breakfast skipping is not significantly associated with lumbar spine BMD.

There are several potential mechanisms that can explain why breakfast skipping may lead to a decrease in BMD. Firstly, breakfast skipping is associated with reduced satiety and changes in appetite, which may result in overeating during the next meal and affect insulin sensitivity (51, 57, 58). By contrast, having breakfast is beneficial for appetite regulation, stabilising blood sugar levels, and increasing insulin sensitivity (59). The skeletal system plays a crucial role in glucose and energy metabolism as an important component of the endocrine system. Among them, osteocalcin is a specific non-collagenous protein that is abundant in osteoblasts and a key determinant of bone formation. Research has shown that there is a significant positive correlation between insulin sensitivity and osteocalcin (60), i indicating that reduced insulin sensitivity can affect bone formation, leading to bone loss. Secondly, breakfast skipping is associated with the overactivity of the hypothalamic–pituitary–adrenal axis, often leading to elevated blood pressure in the morning (61). Research suggests a significant association between elevated blood pressure and alterations in calcium metabolism, involving increased movement of calcium from the bones, accelerated calcium loss, and compensatory activation of the parathyroid gland. Furthermore, the detrimental effects of elevated blood pressure on calcium balance are persistent, leading to further reductions in BMD (62). Conversely, eating breakfast has been shown to help lower blood pressure (63), thus reducing the adverse effects associated with elevated blood pressure. Finally, skipping breakfast may also have adverse effects on lipid levels, such as elevated total cholesterol and low-density lipoprotein cholesterol concentrations (11, 52). This has been confirmed as a risk factor for low BMD (18, 64).

However, in our study, we did not find any correlation between breakfast skipping and decreased BMDs. The disparities in inconsistent results can primarily be attributed to the type and design of the research. Previous study conclusions were based on the outcomes of observational studies, and establishing causality is a limitation of observational research. Observational studies are weaker in controlling confounding factors compared with MR analysis. In observational studies, confounding factors cannot be entirely eliminated even when rigorous normalisation processes have been implemented (16). Furthermore, our study used a greater number of instruments, offering enhanced robustness while analysing data, under the assumption of meeting the three core MR assumptions. This methodology enabled a more precise assessment of the relationship between breakfast skipping and BMDs. Lastly, differences in the study populations must be taken into account. Previous reports regarding the correlation between breakfast skipping and BMD mainly focused on Asian populations, in which racial and lifestyle differences from the populations studied here may have produced different findings.

This study had several important strengths. To the best of our knowledge, our study is the first MR study to investigate the causal relationship between breakfast skipping and BMD using GWAS data. Undoubtedly, our MR analysis surpasses previous observational studies as we used summary data from GWAS, providing a significantly larger sample size and a substantial number of SNPs. Importantly, our study yielded robust and reliable results, as there was no evidence of heterogeneity or pleiotropic effects. However, this study still had some limitations. Firstly, the study included populations from Europe and the Americas, so our conclusions should be cautiously extrapolated to other populations. Secondly, our study did not include data from indigenous populations. In future research, it would be necessary to further enhance the availability of GWAS databases for smaller regional areas to provide richer data for comparisons.

## Conclusion

We investigated the causal relationship between skipping breakfast and BMD using MR. The study showed that there was no causal effect of breakfast skipping on BMD at the forearm, femoral neck, or lumbar spine. Further analysis of age-stratified BMD data did not reveal any causal effect of breakfast skipping on BMD. These findings are inconsistent with many previously published studies. When larger-scale GWAS data are available or more advanced methods are proposed, we will reanalyse the data to confirm our research findings, thereby reducing bias and providing more accurate results.

## Data availability statement

Publicly available datasets were analyzed in this study. This data can be found here: https://msk.hugeamp.org/datasets.html http://www.gefos.org/.

## Author contributions

JY: writing-original draft. KT, CZ and WG: conceptualization: project administration: and writing review and editing. XZ, YC and LW: data curation and methodology. RZ and KT: formal analysis: validation: visualization. WL and YZ: software. All authors contributed to the article and approved the submitted version.
